# Deep Learning Based Underwater Acoustic Target Recognition: Introduce a Recent Temporal 2D Modeling Method

**DOI:** 10.3390/s24051633

**Published:** 2024-03-02

**Authors:** Jun Tang, Wenbo Gao, Enxue Ma, Xinmiao Sun, Jinying Ma

**Affiliations:** 1 School of Civil Engineering, Tianjin University, Tianjin 300072, China; tangjun@tju.edu.cn (J.T.); gaowenbo_2022@tju.edu.cn (W.G.);; 2 School of Electrical and Information Engineering, Tianjin University, Tianjin 300072, China; 3 School of Electronic Engineering, Tianjin University of Technology and Education, Tianjin 300222, China

**Keywords:** underwater acoustic target recognition, deep learning, temporal 2D modeling, short-time Fourier transform

## Abstract

In recent years, the application of deep learning models for underwater target recognition has become a popular trend. Most of these are pure 1D models used for processing time-domain signals or pure 2D models used for processing time-frequency spectra. In this paper, a recent temporal 2D modeling method is introduced into the construction of ship radiation noise classification models, combining 1D and 2D. This method is based on the periodic characteristics of time-domain signals, shaping them into 2D signals and discovering long-term correlations between sampling points through 2D convolution to compensate for the limitations of 1D convolution. Integrating this method with the current state-of-the-art model structure and using samples from the Deepship database for network training and testing, it was found that this method could further improve the accuracy (0.9%) and reduce the parameter count (30%), providing a new option for model construction and optimization. Meanwhile, the effectiveness of training models using time-domain signals or time-frequency representations has been compared, finding that the model based on time-domain signals is more sensitive and has a smaller storage footprint (reduced to 30%), whereas the model based on time-frequency representation can achieve higher accuracy (1–2%).

## 1. Introduction

Acoustic signals are the only energy form known to humans that can travel long distances underwater and are generally considered to be the best information carrier for underwater targets [1]. So, for a long time, underwater acoustic target recognition (UATR) technology has been an important auxiliary tool for marine resource development, playing an important role in both civilian and military applications. Affected by subsea reverberation [2], multipath effects [3], Doppler effects [4], etc., collected ship radiated noise is always accompanied by interference noise. Traditional UATR technology mainly relies on signal processing methods to reduce interference [5], extract features characterizing ship attributes [6,7], and design classifiers for recognition [8]. There are two difficulties: it is easy to eliminate the target signal information while filtering out some strong interference; with the upgrading of marine equipment, the interference is becoming increasingly sophisticated, making it difficult to find a universal feature extraction method [9]. Deep neural networks (DNNs) provide new ideas for researchers that can achieve mapping from acoustic signals to category labels by continuously iterating to fit the nonlinear function and be the classifier to achieve UATR [10].

**Figure 1:**
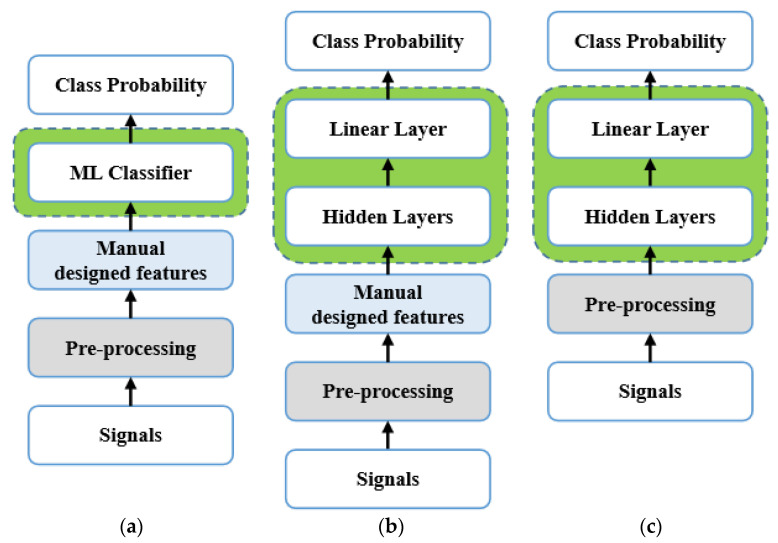
Various UATR pipelines, (**a**) is based on Machine Learning (ML), (**b**) is based on DNN adopting pattern recognition mode, (**c**) is also based on deep learning but adopting end-to-end pattern.

The pipelines of UATR based on Machine Learning (ML) or DNNs are shown in Figure 1, which mainly includes pre-processing, feature extraction, and classifier design. Pre-processing generally refers to audio down-sampling [11,12] and amplitude normalization [13,14]. Signal processing methods are also applied, and some specific filters are designed to meet the research needs [15] or improve signal quality [16,17,18]. Regarding features for classification and recognition, the power spectrum was often used in early research [19]. Detection of envelope modulation on noise (DEMON) and low-frequency analysis and recording (LOFAR) are commonly used spectral analysis methods as manually designed features in UATR [20,21,22,23]. Constant-Q transform (CQT) [24], Mel frequency cepstral coefficients (MFCC) [25], and Gammatone frequency cepstral coefficients (GFCC) [26] also perform well which simulate the auditory perception of human ears. In addition, some studies used multiple spectra [14] or information from multiple modalities [12] to obtain the fusion feature of targets, which is helpful for improving robustness. Recently, neural networks are also widely used in experiments to further extract features and compress feature spaces [10]. In addition to first transforming raw and non-stationary time domain signals into the frequency domain and then inputting them to the network, it is feasible to directly input time domain signals, which treat signals as time series or text sequences. This approach captures the correlation between sampling points and mines inter-class features using hidden layers of the network model [27,28,29].

1D convolutional layers and recurrent neural network layer layers are two kinds of suitable layers to process time series. Inspired by residual networks (ResNets), Doan et al. introduced and designed a 1D dense convolutional neural network that replaces the addition operation in the original skip-connection technique with concatenation [13]. Tian et al. proposed a multi-scale residual unit to generate feature maps with multiple resolutions and avoid the inadequacy problem of small convolutional kernels [11]. Xue et al. added the channel attention mechanism to their model to extract more useful feature information [15]. Yang Jirui, et al. improved channel attention mechanism to better adapt to the characteristics of underwater acoustic signals [17]. However, both 1D convolution and RNNs can only model changes between adjacent time points, thus failing to discover long-term dependencies of sequences. Unlike the above works, Kamal et al. used a set of bandpass filters to divide the raw signal into multiple frequency band components and then merged these components into a 2D tensor, which can be input for 2D convolution [30]. Hu et al. adopted another 2D-variation method that combined the 1D feature vectors of multiple channels and converted the vectors into 2D tensors, then used several 2D time-dilated convolutional blocks to capture deep features for classification [31].

Inspired by the periodicity of natural time series, such as changes in rainfall during the rainy and dry seasons, a new temporal 2D-variation model is proposed and performs well in some time series analysis tasks in [32]. The modeling method can be summarized as follows: using frequency domain characteristics of time series to discover periods, then shaping the time series into a stack of multiple periods, which transforms raw 1D temporal signals to a set of 2D tensors. The obtained 2D tensors contain intraperiod and interperiod variations of the signals. Considering that underwater radiation noise of ships usually has obvious periodic characteristics, in this paper, we introduce a recent 2D modeling method into UATR to combine conv1D and conv2D, and use Timesblocks to capture periodic features of underwater acoustic time domain signal on the basis of current convolutional neural networks. Compared to current research that focuses solely on 1D or 2D convolution, our approach not only prevents information loss during time-frequency conversion and overcomes the limitations of pure 1D convolution, but also provides a new idea for model structure design. Our work mainly includes the following parts:

- We train models using time domain signals and time-frequency representations, obtain a network structure suitable for UATR, and analyze the performance and applicable scenarios of these two types of inputs.
- We adopt a recent temporal modeling method to transform the time-domain feature vectors of underwater acoustic signals extracted in 1D convolution into 2D tensors, and then use 2D convolution to further extract periodic characteristics. By adding Timesblocks to two excellent model structures, the models break through bottlenecks in the original structure’s recognition ability.

The remainder of this paper is organized as follows. The 2D modeling method and Timesblock are described in detail in the Methods section. The used dataset, data pre-processing methods, comparison models, and training strategy were introduced in the experiment. The results show the experimental results and some discussion. Finally, the Conclusion section presents a summary of this paper.

## 2. Methods

### 2.1. Temporal 2D-Variation Modeling

**Figure 2:**
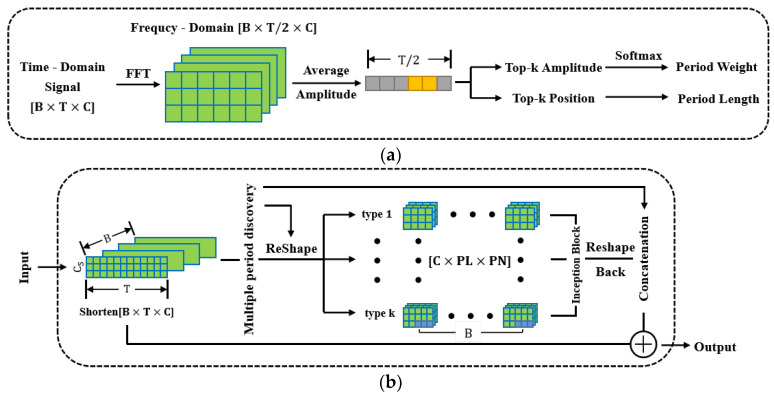
Partial structure of TimesNet: (**a**) Algorithm diagram to discover multi periods; (**b**) Structure of Timesblock, which performs the transition from 1D→2D→1D, and reshapes (2D modeling) and reshapes back represent from 1D→2D and from 2D→1D respectively.

The core of the 2D modeling method is discovering the periodicity of the time series, which is achieved by searching for a series of periods. As shown in Figure 2a, the algorithm can be divided into the following five steps.

- Perform a fast Fourier transform (FFT) on the time series $X_{1D}$ to convert it into a frequency domain sequence $Y_{1D}$. Only retain the first half of the frequency domain sequence because the result obtained by FFT is middle-symmetric. The dimension of the original series is T×C, where T is the length of the time series and C is the number of channels.
- Calculate the average amplitude $Amp_{Y_{1D}}$ of the frequency domain sequence $Y_{1D}$ for all channels. The first amplitude is set to 0 considering the characteristics of FFT.
- Assuming that the raw time series $X_{1D}$ has k types of periods, and each period has a different length, record the Top-k + 1 amplitudes $\left\{Amp_{f_{1}},\;\ldots ,\;Amp_{f_{k+1}}\right\}$ and the corresponding positions $\left\{p_{f_{1}},\;\ldots ,\;p_{f_{k+1}}\right\}$. Some modifications are made here, which are summarized in subsequent experiments.
- The position is considered as the period length, and the number of periods in a signal sequence is $\mathrm{Ceil}\left\{\frac{T}{p_{f_{2}}},\ldots ,\frac{T}{p_{f_{k+1}}}\right\}$, where Ceil is the rounding up operation, ensuring that all sampling points are counted.
- Normalize amplitudes $\left\{Amp_{f_{2}},\;\ldots \;,\;Amp_{f_{k+1}}\right\}$ using the softmax function to obtain weights $\left\{\omega_{1},\;\ldots ,\;\omega_{k}\right\}$ that represent the importance of each period.

In summary, multiple periods of the series are discovered based on the frequency domain characteristics, and different periods are assigned corresponding weights.

### 2.2. TimesNet and Timesblock

TimesNet is a neural network that accepts general time series. First, a 1D convolution layer is used as token embedding to adjust the number of channels, and the position embedding method in Transformer [33] is adopted to record sequential information of time points. The results of embedding are passed through the dropout layer to prevent overfitting. Next, extract periodic features of each channel through several stacked Timesblocks which are shown in Figure 2b. The output of each Timesblock was normalized to accelerate convergence. After activation through the activation function geLU, the obtained multichannel feature matrix was expanded into a long feature vector. Finally, the vector is compressed through one linear layer, and the softmax function is used to calculate the probability of belonging to a certain category.

TimesBlock is the backbone and the most critical component of TimesNet, which can extract spatiotemporal 2D features of intraperiod and interperiod variations after 2D modeling. The feature extraction process of TimesBlock is as follows:

- Based on the temporal 2D-variation modeling mentioned in Section 2.1, *k* types of periods and weights of the input signal can be calculated.
- Transform the raw time series into a set of 2D tensors {$X_{2D}^{i}\in R^{L_{i}\times N_{i}\times C}$}, where $L_{i}$ is the i-th period length, indicating that each column contains the time points within one period; $N_{i}$ is the number of the i-th period, representing that each row contains the time points at the same phase among different periods. To add, {$\frac{T}{p_{f_{i}}}$} is often not an integer, which means that the number of sampling points in the last period is less than the period length. So, to obtain a complete 2D tensor, it is necessary to padd zero for most signal sequences before reshaping.
- Input these tensors into two inception blocks in series that contain multiple-scale convolutional kernels to extract feature maps of intraperiod and interperiod variations.
- Reshape the extracted feature maps back into 1D feature vectors, removing the previously filled tails.
- Calculate the weighted average feature vectors for all periods, with weights derived from the algorithm in the first step.
- The final feature vector, i.e., the output of one Timesblock, is obtained by adding the weighted average feature vector from the previous step as a residual to the original series.

## 3. Preparation

### 3.1. Data Source

There are two publicly available underwater sound databases to choose from: ShipsEar [34] and Deepship [35]. In past research, these two databases have been widely used, and their reliability in classification has been demonstrated. The ship radiated noise in ShipsEar is divided into four major categories based on the size of the ship. Deepship contains four classes of ships: Cargo, Passenger, Tanker, Tug, each with a longer duration. Compared to ShipsEar, the records in Deepship have complex background components, which results in a low signal-to-noise ratio (SNR) and the ability to more clearly verify the effectiveness of classification methods.

### 3.2. Dataset Manufacture

Regarding the manufacturing of training and testing samples, research on classification based on underwater acoustic time-domain signals (T) usually divides the entire audio into a certain number of frames, each frame containing approximately thousands of sampling points (less than 1s in duration), whereas features are often extracted from longer segments (approximately a few seconds) in research based on time-frequency representation (T-F). The basis for the first is that compared to the background, target features are relatively stable in each frame and can be captured by deep learning models, which can also obtain a larger number of samples and reduce memory usage during training. The second is subjectively more reasonable, just as sonar men need a period of listening to complete recognition tasks. Here, we consider these two methods and attempt to compare them.

**Figure 3:**
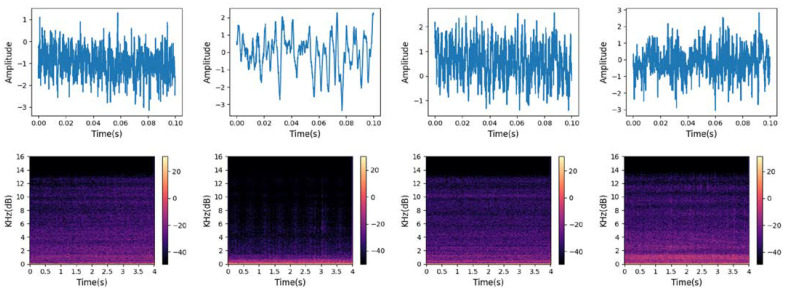
Waveform (above, frames with durations of 0.1 s) and T-F representation (below, segments with durations of 4 s) of the four ship categories. From left to right are Cargo, Tanker, Tug, and Passenger. The sampling points were standardized.

Due to the fact that most of the characteristics of ship-radiated noise are concentrated in low-frequency components, audio is down-sampled to 8000 Hz and then divided into frames and segments. There were 4000 sample points for each frame and no overlap between frames. Meanwhile, each segment has a duration of four seconds, and short-time Fourier transform (STFT) is performed to obtain the corresponding time-frequency feature maps whose size is 256×256. Some tails that were not long enough were removed, and the results are shown in Table 1. Figure 3 shows the waveform and T-F representation of each category. It can be observed that there are significant differences in the amplitude, amplitude fluctuation, and local extreme frequency of the waveform. In addition, the low-frequency frequency band components of ship-radiated noise vary greatly among different categories. These reflect the separability between categories.

### 3.3. Protocol

The entire experiment was divided into two parts. Experiments to compare the performance of models based on time-domain signals or time-frequency representation were first conducted. Another main purpose of the experiment was to prove the validity of the recent 2D modeling method. Considering that TimesNet was originally designed to handle typical time series analysis tasks, and compared to general time series classification tasks, ship radiated noise samples have a higher sampling density, contain more sampling points, and have significant background interference, making it difficult for the original TimesNet to play an effective role. Therefore, we decided to add TimesBolcks to outstanding model architectures in an attempt to gain stronger performance.

Several classical deep-learning architectures with excellent performance, which are state-of-the-art and have been validated in the field of underwater acoustics, are selected to build recognition models, including ResNet [36], SE ResNet [37], CamResNet [15], DenseNet [13], and MSRDN [11]. The information for each model is described briefly below.

- ResNet is a very famous neural network, which responds well to degradation and greatly eliminates the difficulty of training neural networks with excessive depth by adding shortcuts. Activation defaults to using reLU.
- SE ResNet adds the squeezing-and-excitation (SE) module [37] to the initial residual block, which mainly includes two linear layers to calculate the weights of different channels to introduce the channel attention mechanism. Activation defaults to using reLU.
- CamResNet uses 1D convolution instead of linear layers to adapt the SE module, and goes further into the attention mechanism by adding a spatial attention module as an independent branch to the SE module to synthesize the signal characteristics in all channels. Activation defaults to using reLU.
- DenseNet is a variation of ResNet by converting skip-connection from addition to concatenation, which performs well on certain datasets. Every block in DenseNet contains three layers and uses an eLU for activation.
- MSRDN is composed of stacked multi-scale residual units that contain four parallel convolutional layers with different kernel sizes to generate and combine feature maps with multiple resolutions. A soft-threshold learning module is added to the top of the units to generate a threshold for every channel by nonlinear transformation and enhancing the effective channel components. The model uses siLU for activation.
- The backbone of each model is a stack of several convolutional blocks, the bottom layer is a convolutional block that adjusts the channel from 1 to the specified number, and the top layers contain one average-pooling, which significantly reduces the number of parameters in the linear layer and effectively prevents overfitting. One linear layer is placed at the end to output the probabilities.

In addition, these models were not completely copied and adjusted during the experimental process because different datasets and data processing methods were used.

The evaluation indicators are based on convention, using an accuracy and confusion matrix. The accuracy reflects the effective recognition ratio of the model to the overall dataset, and the confusion matrix focuses on a local level that contains the precision and recall of each class. Due to the relatively balanced number of samples in each class within the dataset, the F1 score has not received much attention. In addition, the parameter quantity and inference time of the models are also recorded as auxiliary evaluation indicators.

The execution of data processing utilizes two Python packages, librosa, to read audio files (. Wav) and perform STFT and sklearn to perform standardization. Training and testing were conducted on a regular rack server with an Nvidia GeForce RTX 3090 GPU (24 G), and the network model was implemented on the open-source ML framework pytorch-2.0.1 under Linux. The arrangement of model training and testing is as follows:

data type = {T or T-F}, batch size = 64, optimizer = Adam, initial learning rate = 0.001, epoch = 50, training:testing = 4:1.

The models were trained from scratch, and the network weights were updated after completing each batch of training. To add, the learning rate is adaptively adjusted, and the adjustment formula is as follows, where the initial ${\mathrm{lr}}_{0}$ is set to 0.001.

$${\mathrm{lr}}_{i+1}={\mathrm{lr}}_{i}\times 0.5^{\mathrm{epoch}-1}$$

## 4. Results and Discussion

**Figure 4:**
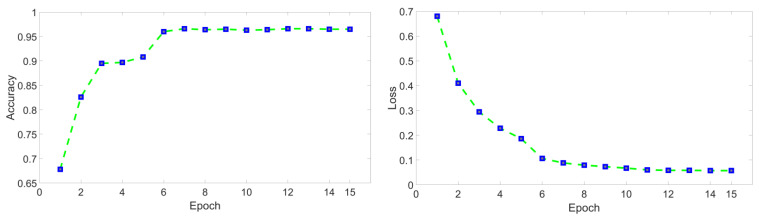
Example of accuracy and loss variation curve. The blue point represents the value of an epoch and it can be found that the convergence of the model was satisfactory.

**Figure 5:**
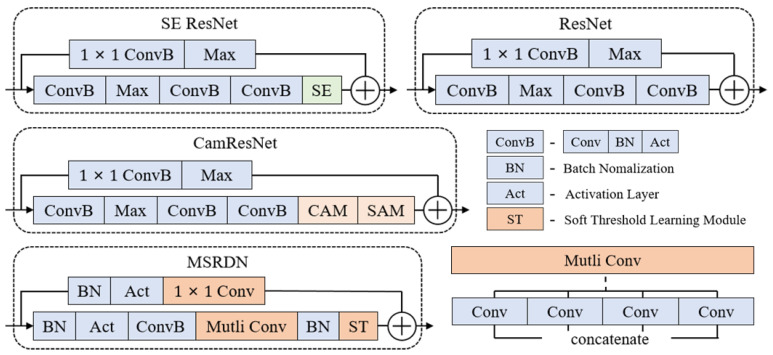
Block structures of each model used in the experiment. CAM represents the channel attention mechanism, and SAM represents the spatial attention mechanism.

**Part I** Models mentioned in Section 3.3 are trained. Each model was fully trained, and the training mostly lasted for 17 to 22 epochs, achieving the highest accuracy rate, as shown in Figure 4. During training, by adding and modifying networks layer by layer, the performance gains from the improvements were gradually reflected. The final structures are shown in Figure 5. Meanwhile, some hyper-parameters in the models are adjusted to achieve the best performance, which are listed in Table 2. The storage size of the parameter file is used to represent the parameter quantity of the model, and the inference time is represented by the average training time of completing a batch. The values of the evaluation indicators are shown in Table 3.

From the overall experimental results, the T-F-models achieved higher accuracy. Due to the same structure in both types of models, this can be attributed to the fact that T-F representations highlight differences between categories more effectively, resulting in better separability. However, DNNs can also rely on short-frame signals to make reliable judgments, which are not inferior. Moreover, T-models have the advantages of a smaller memory footprint and faster response time with almost no need for preprocessing units, which makes them more suitable for deployment on edge detection devices with insufficient storage space. During the experiments, it was also found that as the number of channels increased, the parameter quantity of the T-F-models using 2D convolution gradually widened the gap with the T-models, as shown in Table 3, and these additional floating-point operations also affected the inference speed. As each model, the SE module is quite powerful, as it improves the accuracy of ResNet without significantly affecting other indicators. CamResNet did not outperform ResNet, which may be due to the lack of robust spatial characteristics of underwater acoustic signals, making it more difficult for the model to converge. Convolutions in the attention mechanism module have a negative effect, which significantly increases the floating-point operand. It was also found that parallel structures in MSRDN can cope with overfitting and provide the possibility of adapting to more complex structures. The unique connection mechanism in DenseNet causes the number of channels to rapidly expand, and continuous pooling causes feature vectors (or maps) to quickly decrease in size, making it difficult to deepen, resulting in poor performance.

**Figure 6:**
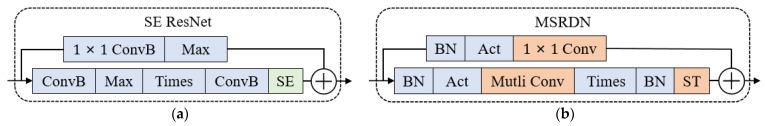
Blocks with Timesblocks. (**a**) SE ResNet and (**b**) MSRDN.

**Part II** The Timesblock is added to the original blocks of SE ResNet and MSRDN, respectively, to replace 1D convolutional for further improvement, and the new structures are shown in Figure 6. In the Timesblock, there is one unique hyperparameter: Top-k k. A set of controlled experiments was conducted to determine the optimal value k for this task. The experimental configuration is as follows:

$k=\left\{1,\;2,\;3\;\mathrm{or}\;4\right\}$, Inception channels $C=\left\{128\;\mathrm{to}\;64\;\mathrm{to}\;128\right\}$, kernel number $n_{k}=\left\{1\;\mathrm{or}\;2\right\}$, and kernel size $m_{k}=\left\{1,\;3\right\}$.

During the experiment, dropout was added at the output of the first convolutional block to deal with overfitting. The results are shown in Table 4, and it can be found that the Timesblock can indeed play a role. To our surprise, introducing Timeblocks can reduce the parameter quantity of the model. Since k is independent of the network layer, its selection only affects the inference speed, and it is not recommended to use a larger k as it does not benefit testing.

**Figure 7:**
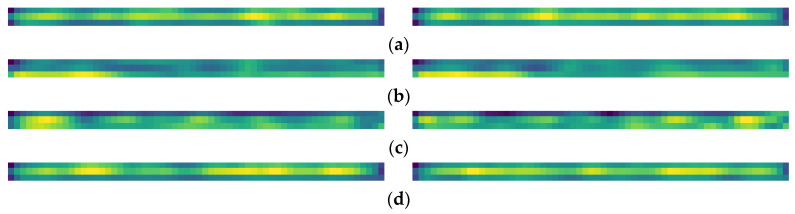
Examples of thermal maps. (**a**–**d**) are Cargo, Tanker, Tug, and Passenger, respectively.

To verify whether the Timesblock captures the multi-periodicity of ship radiated noise, heat maps of feature matrices extracted from the last Timesblock are drawn. Figure 7 shows some cases from which it can be seen that the transformed 2D tensors are informative and clearly distinguishable.

**Figure 8:**
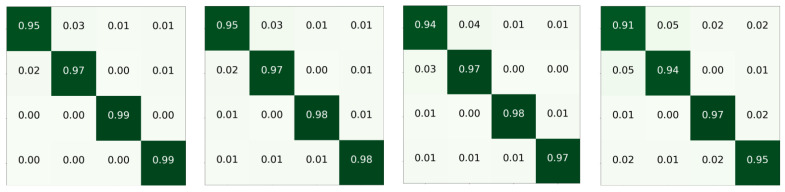
Confusion matrices. The darker the green, the higher the probability. The first to fourth rows are Cargo, Tanker, Tug, and Passenger, respectively, and from left to right are T-F-MSRDN, T-F-SE ResNet, T-SE ResNet with Timesblocks, and T-MSRDN with Timesblocks.

**Part III** Specific to each category, some confusion matrices are shown in Figure 8, and the detailed data of the confusion matrix obtained by SE ResNet with 2D modeling is shown in Table 5. It can be seen that the passenger is easy to identify, which has the highest recall and precision, while Cargo and Tanker are prone to confusion. After manually listening to audio, we speculate that the cause of confusion may be that such ships do not have relatively consistent rotational frequencies and axial frequencies. In addition, considering that the SNR of audio in Deepship is relatively low, some frames may be submerged by interference, which affects the training of the model.

**Figure 9:**
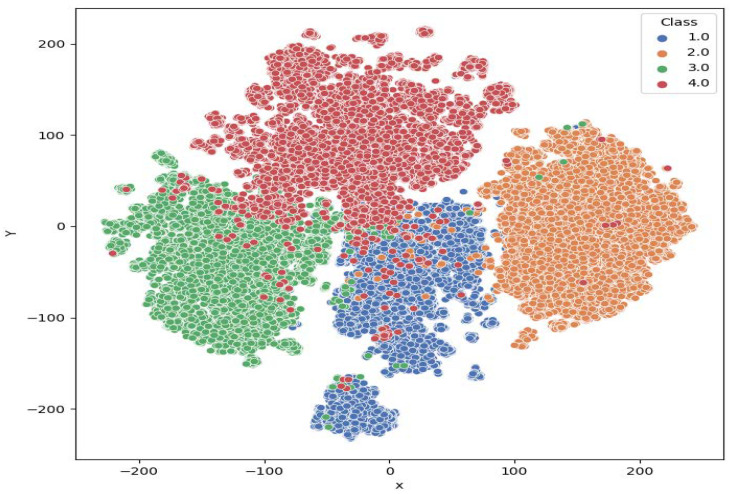
t-SNE visualization. Class 1.0 to 4.0 are Cargo, Tanker, Tug, and Passenger, respectively.

In order to examine the model performance and show the experimental results more intuitively, the t-distributed Stochastic Neighbor Embedding (t-SNE) [38] method was used to visualize the target features extracted from the model. As shown in Figure 9, different categories of features extracted are basically separated, and the main intersections are concentrated at the boundaries of Cargo and Tanker, which is consistent with what is reflected in the confusion matrix.

## 5. Conclusions

The impact of two types of inputs (time domain waveform or time-frequency representation) on the underwater acoustic target recognition model has been analyzed. In contrast, models based on time-frequency representation can achieve higher accuracy (1–2%), while including more network parameters and floating-point operations, which is more evident when networks become complex; models based on time domain signals have faster inference speed and model files are smaller, making them suitable for deployment on edge devices with insufficient computing power and limited memory.

SE ResNet and MSRDN are two high-performance model structures. This paper introduces a recent temporal 2D modeling method from TimesNet into these structures. The modeling method tries to find multiple periods of 1D time domain signals based on frequency domain characteristics, and on this basis, transforms the signal $X_{1D}\in R^{T\times C}$ into a set of 2D tensor $\left\{X_{2D}^{i}\in R^{L_{i}\times N_{i}\times C},\;i\in \left(1,\;k\right)\right\}$. Then, 2D convolution blocks can play a role in capturing features of intraperiod- and interperiod- variations of signals. By adding Timesblocks, recognition rates of models in real underwater acoustic experiments can be improved (0.7–0.9%).

By analyzing the situation of samples in Deepship based on confusion matrices, find that identifying cargo is difficult, while identifying Tug is easier, which can be demonstrated by visualization results of feature vectors of each category of test samples using t-SNE.

## Figures and Tables

**Table 1:** Dataset description.

	Class				Total
	Cargo	Tanker	Tug	Passenger	
Duration (s)	33,530	38,346	35,392	38,609	145,877
Number of Frames	67,060	76,692	70,784	77,217	291,753
Number of Segments	9580	10,956	10,112	11,031	41,679

**Table 2:** Hyperparameters of most models. The multi-scale convolution kernel size of the 1D MSRDN follows the setting in Ref. [11], and the 2D version adopts the settings of the second group in this table. The number of blocks was set to 3 in DenseNet.

**Hyper-Parameters**	**Considered Values**	**Best Values**
Kernel Size 1D $k_{1}$	7, 11, 15, 19, 21, 23	21
Kernel Size 2D $k_{2}$	3, 5, 7, 9	7
Channels C	32, 64, 128, 196, 256	128
Number of Blocks *n_b_*	2, 3, 4, 5	4
**Hyper-Parameters**	**Selected Value**	
Multi-Scale Kernel *m_k_*	$\left\{3,\;9,\;15,\;21\right\}$, $\left\{1,\;3,\;5,\;7\right\}$	
Stride *s*	1, 2 for MSRDN	
Max pooling	kernel size = 3, stride = 2	

**Table 3:** Experimental results for two types of inputs.

Input	Model	Accuracy (%)	Params (M)	Avg. Time (s)
**T**	ResNet	93.76	16.09	0.0495
	SE ResNet	95.71	16.98	0.0506
	CamResNet	93.53	136.75	0.1841
	MSRDN	93.99	4.12	0.0432
	DenseNet	91.47	62.42	0.0557
**T-F**	ResNet	96.33	37.11	0.4003
	SE ResNet	96.86	37.37	0.4032
	CamResNet	95.72	352.93	1.3394
	MSRDN	97.59	6.38	0.3531
	DenseNet	93.32	656.90	0.4226

**Table 4:** Experimental results for the time blocks.

Model	Timesblock	Accuracy (%)	Params (M)	Avg. Time (s)
**SE ResNet**	-	95.71	16.98	0.0506
	*k* = 1, *n_k_* = 1	95.81	11.41	0.0585
	*k* = 1, *n_k_* = 2	96.62	14.23	0.0744
	*k* = 2, *n_k_* = 2	95.92	14.23	0.1110
	*k* = 3, *n_k_* = 2	96.10	14.23	0.1343
**MSRDN**	-	93.99	4.12	0.0432
	*k* = 1, *n_k_* = 1	94.31	4.11	0.0422
	*k* = 1, *n_k_* = 2	87.27	6.37	0.0423
	*k* = 2, *n_k_* = 1	93.26	4.11	0.0431
	*k* = 2, *n_k_* = 2	93.33	6.37	0.0436
	*k* = 3, *n_k_* = 1	94.21	4.11	0.0432
	k = 4, $n_{k}$ = 1	94.36	4.11	0.0433

**Table 5:** Confusion matrix.

True	Predicted				
	Cargo	Tanker	Tug	Passenger	Recall
**Cargo**	12,665	380	175	192	0.944
**Tanker**	434	14,868	21	16	0.969
**Tug**	139	14	13,879	125	0.980
**Passenger**	169	160	148	14,967	0.969
**Precision**	0.945	0.964	0.977	0.978	0.966

## Data Availability

Publicly available datasets were analyzed in this study. The [34,36] mention the acquisition address of the dataset.

## Abbreviations

The following abbreviations were used in this paper:

UATR	underwater acoustic target recognition
DNN	Deep Neural Network
ML	machine learning
DEMON	Detection of envelope modulation on noise
LOFAR	Low-frequency analysis and recording
CQT	Constant-Q transform
MFCC	Mel frequency cepstral coefficients
GFCC	Gammatone frequency cepstral coefficients
SNR	signal-to-noise ratio
STFT	short-time Fourier transform
t-SNE	t-Distributed Stochastic Neighbor Embedding
